# Finding reproducible cluster partitions for the k-means algorithm

**DOI:** 10.1186/1471-2105-14-S1-S8

**Published:** 2013-01-14

**Authors:** Paulo JG Lisboa, Terence A Etchells, Ian H Jarman, Simon J Chambers

**Affiliations:** 1 School of Computing and Mathematical Sciences, Byrom Street, Liverpool John Moores University, Liverpool L3 3AF, UK

## Abstract

K-means clustering is widely used for exploratory data analysis. While its dependence on initialisation is well-known, it is common practice to assume that the partition with lowest sum-of-squares (SSQ) total i.e. within cluster variance, is both reproducible under repeated initialisations and also the closest that k-means can provide to true structure, when applied to synthetic data. We show that this is generally the case for small numbers of clusters, but for values of k that are still of theoretical and practical interest, similar values of SSQ can correspond to markedly different cluster partitions.

This paper extends stability measures previously presented in the context of finding optimal values of cluster number, into a component of a 2-d map of the local minima found by the k-means algorithm, from which not only can values of k be identified for further analysis but, more importantly, it is made clear whether the best SSQ is a suitable solution or whether obtaining a consistently good partition requires further application of the stability index. The proposed method is illustrated by application to five synthetic datasets replicating a real world breast cancer dataset with varying data density, and a large bioinformatics dataset.

## Background

Structure finding in large datasets is important in first line exploratory data analysis. Clustering methods are commonly used for this purpose and among them k-means clustering is widely used.

This has led to variations such as penalised and weighted k-means [1] and in combination with other methods [2]. More complex approaches include hybrid hierarchical k-means clustering algorithms with fewer parameters to adjust [3]. All of these methods depart considerably from the standard implementations that are still of interest.

Practical applications may require the derivation of a partition of the data that is representative of the best achievable clustering performance of the algorithm. This solution should clearly be reproducible under repeated initialisations. However, it is well known that the k-means algorithm has guaranteed convergence only to a local minimum of a sum of squares objective function [4], [5], [6], finding the global optimum is in general, NP-hard [7]. It is commonly assumed that it is sufficient to carry out a number of random initialisations followed by selection of the best separated solution, for instance measured by the sum of square distances from cluster prototypes (SSQ). Various aspects of this process, from the choice of separation measures to the best number of clusters, k, are guided by heuristic methods, reviewed in more detail later in this section.

It is perhaps surprising that as widely used a method as the standard k-means algorithm does not have published, to our knowledge, a systematic assessment of whether it is always the case that the best SSQ suffices. We will show that while this is generally the case, as the number of clusters, k, increases within a range of practical interest, solutions with SSQ very close to the optimal value for that value of k can be substantially different from each other. This requires identification of a procedure to draw a single partition set which is both well-separated and stable, in the sense that a very similar solution will be found by repeating the whole procedure from the beginning.

This is not to say that there is a unique best solution. Rather, the intention is to reproduce convergence to within a set of data partitions which is mutually consistent and similarly well associated with the data structure. The test that is applied is to measure the internal consistency of the clustering solutions obtained for a given value of k. A synthetic data set will be used to evaluate the reliability of the proposed method.

Recent comprehensive reviews of the k-means algorithm [4], [5], [6] do not describe any prescriptive method that is empirically demonstrated to consistently return a clustering solution well associated with the known data structure, nor do they confirm the reliability of the method empirically by repeated mechanistic application to the same data set.

Historically, the motivation for the k-means algorithm included minimising variance in stratified sampling [8,9].This defines a sum-of-squares objective function consisting of the within-cluster variance of the sample, for which a convergent algorithm was later defined [10] by iterating two main stages - definition of prototypes and allocation of continuous data to each prototype. The prototypes assume special status by virtue of achieving a local optimum for the sample variance. Further optimisation beyond convergence of the batch algorithm is possible using on-line updates [11]. This is the clustering method used throughout in this paper. These two stages remain the core of generalised k-means procedures [4]. They were linked to maximum likelihood estimation by Diday and Schroeder [4] and, for model-based clustering, can be optimised with the EM algorithm [12]. The structure of the method can also be extended with the use of kernels [13] and it can be also applied to discrete data [14].

The ill-posed nature of the optimisation task in the standard algorithm[4-6] invites more specific guidance in the selection of solutions. An obvious first step is to choose the solution with the best empirical value of the objective function. The link with maximum likelihood points to non-optimality of the global minimum of the objective function for the purpose of recovering the underlying structure of the data, consistent with the finding that simple minimization of the objective function can lead to sub-optimal results [15,16].This has led to departures from the standard algorithm to specify the choice of initial prototypes [16] and to impose order relationships on the data using adjacency requirements [15]. When attempting to sample a single partition set from the numerous local minima, two indicators may be of use. The first one is to measure the separation between clusters, which relates to a diagnostic test for when a partition set can be trusted [16].

The second indicator is the stability of cluster partitions in relation to each other [17].This is the closest work to the proposed method, albeit focusing on the choice of number of prototypes, k, with the conjecture that the correct number of clusters will result in an improvement in between-cluster stability, estimated by sub-sampling the data. While we query the definition of the consistency index from a statistical point of view, we have verified this conjecture when applied to multiple random initialisations of the complete data set and suggest this approach as a guide to the choice of cluster number, reflecting the view that ''when dividing data into an optimal number of clusters, the resulting partition is most resilient to random perturbations" [4]. This work is among several relating to the application of stability measures to clustering [18,19] but they do not provide specific guidance for the selection of partitions.

The paper therefore proposes and evaluates a practical and straightforward framework to guide the application of k-means clustering, with the property that multiple applications of the framework result in very similar clustering solutions with clearly defined optimality properties, even in the presence of complex data structures containing anisotropic and contiguous clusters, as well as high dimensional data. Application of the framework to the datasets gives a measure of the performance of the framework. To this aim, we specify the task to be the selection of a partition of the data i.e. into non-overlapping subsets, given a value of 'k' for the number of prototypes, by repeated application of the standard k-means algorithm. Therefore, this paper does not address ensemble and consensus methods or model-based extensions of the standard algorithm. Neither do we discuss the relative merits of different data representations by pre-processing, including dimensionality reduction and the choice of distance measure all of which strongly condition the solution space. These approaches represent deviations from the standard algorithm [11], and so are outside the scope of the current paper.

The motivation for seeking a representative, stable clustering solution, with the standard k-means algorithm is the observation, from the decomposition of the objective function in terms of the principal eigenvalues of the data covariance matrix, that near-optimal solutions may be found, in which case they will be stable in the sense that any other 'good' partitions will be 'close' to them [7]. Yet, we show with our empirical results that the quality of clustering solutions can vary substantially both in measures of cluster separation and in the consistency from one random initialisation to the next. This is the case even when considering only well separated solutions.

From a more practical perspective, k-means based algorithms are commonly used in the sub-typing of diseases [20] or for the analysis of DNA microarray data [21], [22], [23], [24] where it is commonly used to allow researchers to gain insights and a better understanding of gene similarity. Of importance in these studies is the stability of the solutions obtained, if the results are unstable, any inferences may change with an alternate run of the algorithm. Use of the framework generates a map of clustering solutions where the appearance of structure reveals the typical variation in cluster performance that can be obtained, offering guidance to when to stop sampling. Most importantly it does so using an efficient approach to stabilise the solutions even when applied to complex and challenging data.

It is important to distinguish between the two objectives: the selection of an appropriate value of **k **and the selection of a stable reproducible solution. The gross structure of the SeCo map of local minima makes a useful indicator of good choices for the assumed cluster number, k. The proposed framework therefore provides an empirical equivalent to the methodology proposed for density-based clustering by [25], however the purpose of this paper is not to evaluate methods for determining the appropriate value of **k**.

More closely related to this paper is the concept of optimality of structure recovery and is naturally expressed in obtaining self-consistency when reproducing solutions. This leads to consideration of the stability of the partitions, for instance to guide consensus clustering [18].

We formulate the following hypotheses:

i. Optimisation of the SSQ objective function by the k-means algorithm with on-line updates can result in significantly different solutions with SSQ values close to empirical minimum.

ii. Filtering SSQ values e.g. best 10% allows the degeneracy of similar SSQ values to be resolved by choosing the individual cluster partition with maximum value of the internal consistency index. This is shown to be a representative solution in the sense that it associates well with the data structure on a challenging synthetic data set; moreover, the solution is reproducible since repeated implementations of this procedure will identify very similar partitions.

Section 2 describes the proposed sampling procedures, section 3 introduces the dataset and results to validate the proposed sampling procedures for synthetic data, comprising a mixture of well-separated and overlapping cohorts of anisotropic multivariate normal distributions.

## Methods

The joint optimisation of within-cluster separation and between-cluster stability requires suitable indices to measure these properties. In principle, any reasonable performance measures may be applied in the proposed framework. This study uses the objective function of k-means, the total within cluster sum of squares, defined as:

(1) $$arg\underset{S}{\text{min}}\overset{k}{\underset{i=1}{\sum }}\underset{x_{j}\;in\;S_{i}}{\sum }{\left(x_{j}-u_{i}\right)}^{2}$$

Where *u_i _*is the mean of the points in *s_i_*.

There are several indices of agreement between pairs of cluster labelling, such as cosine similarity and the Jaccard coefficient [17].In the statistical literature there are also inter-rater agreement measures for known labels, such as Cohen's Kappa index. In order to avoid the need for an oracle to set a nominally correct number of clusters [26] a generic index of association, of concordance, must apply to data partitions whose inherent labels are not known in advance, should be normalized and not strongly dependent on the marginal frequencies in each cluster partition [27] and preferably apply to comparisons between partitions with different numbers of clusters. A suitable measure is the Cramérs V-index of concordance [28]. This is a statistical test score to measure the strength of association between two data partitions of the same data set. For a cross-tabulation of *n *observations representing a partition into *p *rows and another as *q *columns, treated as a contingency table with expected entries *E_pq _*for independent cluster allocations and observed values *O_pq_*, the extent to which one cluster partition predicts the other (i.e. the association between them) is measured by

(2) $$C_{v}=\sqrt{\frac{\chi^{2}}{N.\text{min}\left(P-1,Q-1\right)}}$$

where,

(3) $$\chi^{2}=\overset{P}{\underset{p=1}{\sum }}\overset{Q}{\underset{q=1}{\sum }}\frac{{\left(O_{pq}-E_{pq}\right)}^{2}}{E_{pq}}$$

An alternative statistical measure of agreement between two partition sets, and also considered, is the Adjusted Rand Index of Hubert and Arabie (ARI-HA) [29]. The measure was adjusted to avoid over inflation due to correspondence between two partitions arising from chance. The Cramérs V-index and ARI-HA have a Pearson correlation coefficient of 0.99, higher than the value 0.95 between the Cramérs V-index and both the unadjusted ARI of Morey and Agresti and the Jaccard index [29].This shows that the two statistical indices are closely related, though not identical, with even better correlation for better correspondence between partitions.

For a given data set, algorithm and assumed number of clusters k, the following methodology is proposed:

i. Apply the cluster partition algorithm to a sample of size ***N_total _***of cluster initialisations, each seeded with k randomly selected points sampled from the full data set i.e. the standard initialisation for k-means

ii. Sort by separation score and select a fraction **f **by ranked score of **ΔSSQ**, defined as the difference between Total Sum of Squares and the Within Cluster Sum of Squares for a particular solution, returning a working sample of cluster partitions N_sample_= N_total_*fin number

iii. Calculate the N_sample_*(N_sample_-1)/2pair wise concordance indices ***C_V _***for the selected cluster partitions and return the median value **med(CV) **of all pair wise concordance indices for each partition

iv. The Separation and Concordance (SeCo) map comprises the 2-dimensional coordinates **(ΔSSQ, med(CV)) **for the selected cluster partitions.

v. Once the landscape of cluster partitions has been mapped using the SeCo map, where there is a spread of solutions with similar **ΔSSQ**, choose the solutions with the highest value of **med(CV)**.

As the assumed number of partitions, *k*, is increased, the map generates a scatter of points with increasing **ΔSSQ**, but with a distribution of ***med(C_V_) ***that shows the stability of each assumed number of clusters, when fitting the data structure within the constraints of the particular clustering algorithm.

In this paper the total number of initialisations was taken to be 500 and through experimentation, presented in the next section, it is demonstrated that only the top decile by separation need be retained, resulting in a working sample size *N_sample _= 50 *for each value of the assumed cluster number *k *for general purposes. These parameters can be varied, the total sample size being required to be sufficient for a clear group structure to emerge among the cluster solutions in the SeCo map, while retaining a small enough fraction of the total initialisations to avoid cluttering the map.

Two additional measures are used to evaluate the performance of each method, Accuracy, the proportion of correctly classified objects, and Affinity, a measure of how often a data point is allocated to a particular cohort. Affinity is therefore a row level indicator and is calculated by taking a set of solutions and for each row determining the highest proportion for which an element is assigned to a particular cohort, the mean value of this forms an overall indicator of how often data points swap cohorts for the whole dataset.

## Results

### Artificial data

The framework was applied to five synthetic datasets, each of which was produced using identical distributions of three variables, with varying density. The largest dataset comprised 10,000 data points, and the smallest 500, with the intention to replicate real-world scenarios where small datasets are used to evaluate larger populations. The data were sampled in sets of 10000, 5000, 2500, 1000 and 500 data points randomly sampled from a mixture of 10 multivariate normal distributions whose mean vectors and covariance matrices are presented in Table 1. The parameter settings were chosen to replicate a real world breast cancer dataset [30], using the three principle separation axes [31], in particular combining separated and contiguous cohorts.

**Table 1:** Means and covariance matrices for generating the components of the artificial dataset.

	Mean			Covariance Matrix (i,j)									
	x	y	z	11	12	13	21	22	23	31	32	33	N
C1	-0.799	-1.011	-3.336	0.336	0.044	0.074	0.044	0.371	0.21	0.074	0.21	0.582	64
C2	-0.441	-0.569	-2.331	0.428	0.06	-0.002	0.06	0.123	0.157	-0.002	0.157	0.648	42
C3	0.649	-0.344	-4.154	0.62	0.023	-0.035	0.023	0.137	0.07	-0.035	0.07	0.446	61
C4	1.077	0.072	-2.815	0.366	-0.002	0.076	-0.002	0.043	0.104	0.076	0.104	0.563	32
C5	-0.39	-0.242	0.256	0.536	0.013	0.031	0.013	0.348	-0.117	0.031	-0.117	0.689	197
C6	-1.358	-0.658	1.639	0.309	-0.06	-0.055	-0.06	0.245	-0.013	-0.055	-0.013	0.532	131
C7	1.261	0.125	0.862	0.323	0.017	0.027	0.017	0.386	-0.06	0.027	-0.06	0.403	163
C8	-0.593	3.024	-0.498	0.776	0.033	0.175	0.033	0.491	0.003	0.175	0.003	0.695	97
C9	0.251	-0.539	-0.53	0.711	-0.025	0.055	-0.025	0.352	-0.081	0.055	-0.081	0.576	106
C10	0.374	-0.267	1.973	0.39	-0.097	0.041	-0.097	0.343	-0.014	0.041	-0.014	0.322	183

**Figure 1:**
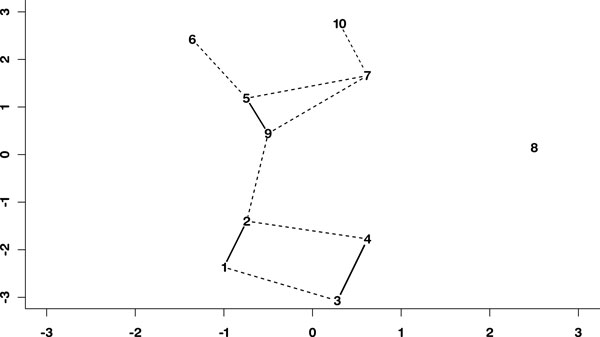
**Sammon projection of synthetic data**. Multidimensional scaling map of the synthetic data using pairwise distances for each of the generating centres of the data. Bold lines indicate mixing of the partitions.

A schematic representation of the mixing structure among the synthetic data cohorts, using a multidimensional scaling [32]to set distances that mirror the pair wise mixing coefficients is shown in Figure 1. The solid lines and dashed lines represent the mixing coefficients highlighted in Table 2.

**Table 2:** Pairwise indices of c-separation for the synthetic data

	C1	C2	C3	C4	C5	C6	C7	C8	C9	C10
C1	0									
C2	**0.7805**	0								
C3	**1.2105**	1.4828	0							
C4	1.5054	**1.1924**	**1.0687**	0						
C5	2.4975	1.7636	3.0649	2.3119	0					
C6	3.3913	2.8294	4.476	3.8029	**1.1757**	0				
C7	3.2516	2.5575	3.7002	2.7302	**1.2151**	2.2233	0			
C8	2.9776	2.4341	3.0901	2.4774	2.025	2.6082	2.2314	0		
C9	2.0388	**1.2969**	2.4543	1.6846	**0.7109**	1.8176	**1.2393**	2.2086	0	
C10	3.7087	3.0487	4.4727	3.5977	1.2717	1.4141	**1.233**	2.5497	1.6952	0

Coefficients <1.30 are highlighted in boldface, those around unity or less underlined

**Figure 2:**
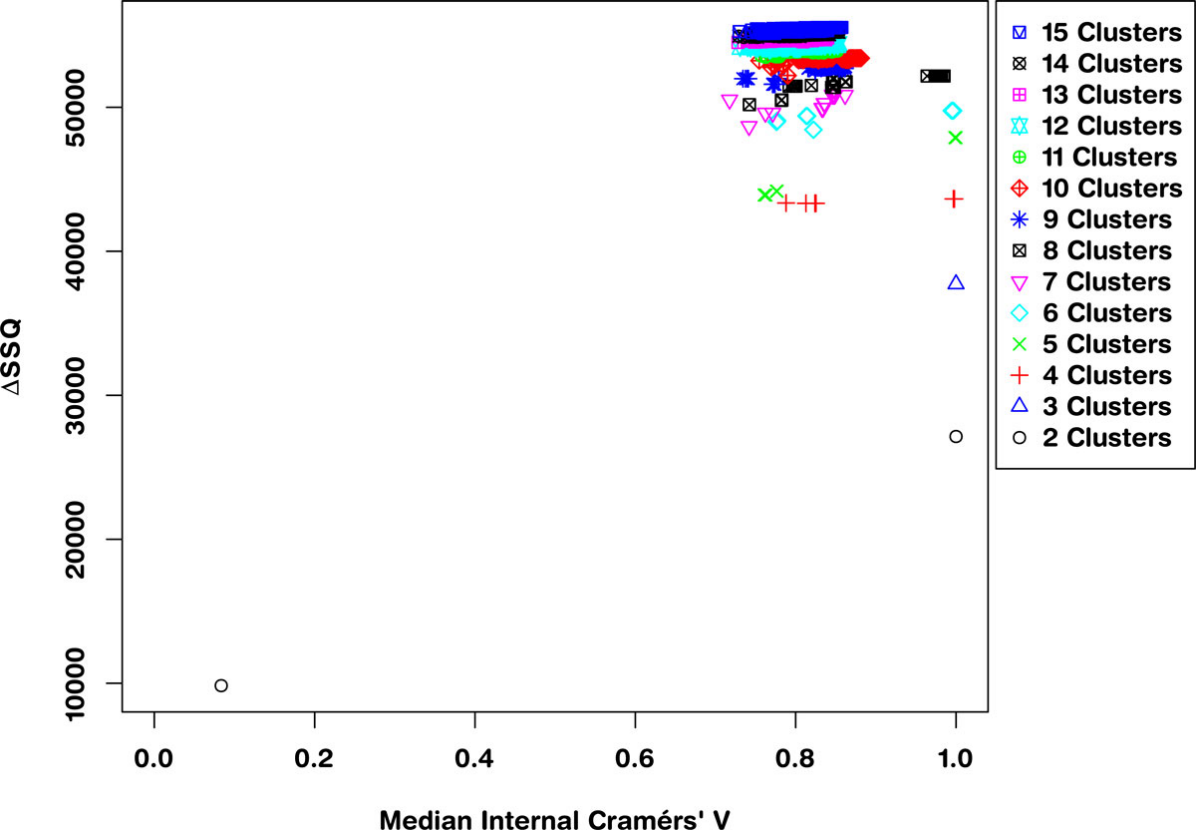
**Separation Concordance map for synthetic dataset**. Full Separation-Concordance map for the synthetic data highlighting the ΔSSQ on the y-axis and the internal median Cramérs' V on the x-axis. For 500 initialisations of the k-means algorithm for each value of **k**.

The sampling of an optimum solution from the conjoint space of separation and concordance is achieved by selecting the solution from the leading right edge of each solution set within the SeCo map. Application of the framework to the 10,000 dataset results in the Separation Concordance map shown in Figure 2 which shows highly aggregated performance for three clusters consistent with segmentation of the data along major axes.

An exact match between the original cohorts and the empirical cluster partitions is not expected because of the mixing and also due to the isotropic Euclidean metric which does not take account of the covariance structure of the data. Therefore instead of the original cohort allocation, k-means was applied to the dataset and repeated 500 times for **k **= 10 and the solution with the lowest SSQ chosen to provide the basis of the reference partition.

The cluster means of this reference partition were then taken, and k-means iterated to convergence on each of the smaller datasets using these centres as the initialisations. These form the reference partitions against which the agreement of empirical k-means solutions with a 'good partition' of the data will be measured.

Applying the framework to the largest of the datasets (10,000 data points) gives a SeCo map, such as that presented in Figure 2. This shows the SeCo map for 500 runs of k-means for numbers of partitions in the range 2 to 15. The y-axis shows the ΔSSQ and the x-axis the Internal Median CV, which is the median of all the pairwise Cramérs' V calculations for each solution.

This SeCo map shows there is a wide range of potential solutions for high values of **k **and that there is substantial variation in values with similar SSQ. As the value of **k **increases, the variation in the solutions increases, and for **k**> = 8, we see a drift to the left on the x-axis as solutions become less and less internally consistent. Even for lower values of **k **there are particular solutions which have much lower median concordance with the other solutions, such as for **k **= 4 and **k **= 5.

### Cardiotocography data

The cardiotocography dataset [33,34] comprises 2126 foetal cardiotocograms for which automatic processing has been applied. These measurements were classified by experts and two consensus classification labels applied to each with respect to morphologic pattern and foetal state. Consequently the data has both three and ten cohorts which could be used to evaluate performance.

Evaluation of this dataset was performed using the 10 class underlying partition however no reference partition was derived as with the artificial data, given the purpose is to evaluate performance in a real-world scenario. This dataset is known to be difficult for the k-means algorithm to correctly partition, and for the ten cohort solution, a previous study has obtained around 40% [35]. For this experiment, the data were scaled to values between 0 and 1, and the equivalent measure of accuracy (mean and standard deviation of the proportion of correctly classified objects over 100 runs) for the single measure (SSQ alone) approach was 37% (s.d. 1.69) and for the dual measure (SSQ, CV) 38% (0.20). For the single and dual metrics the Cramérs' V values were 0.45 (s.d. 0.0103) and 0.46 (s.d. 0.0048) respectively.

**Figure 3:**
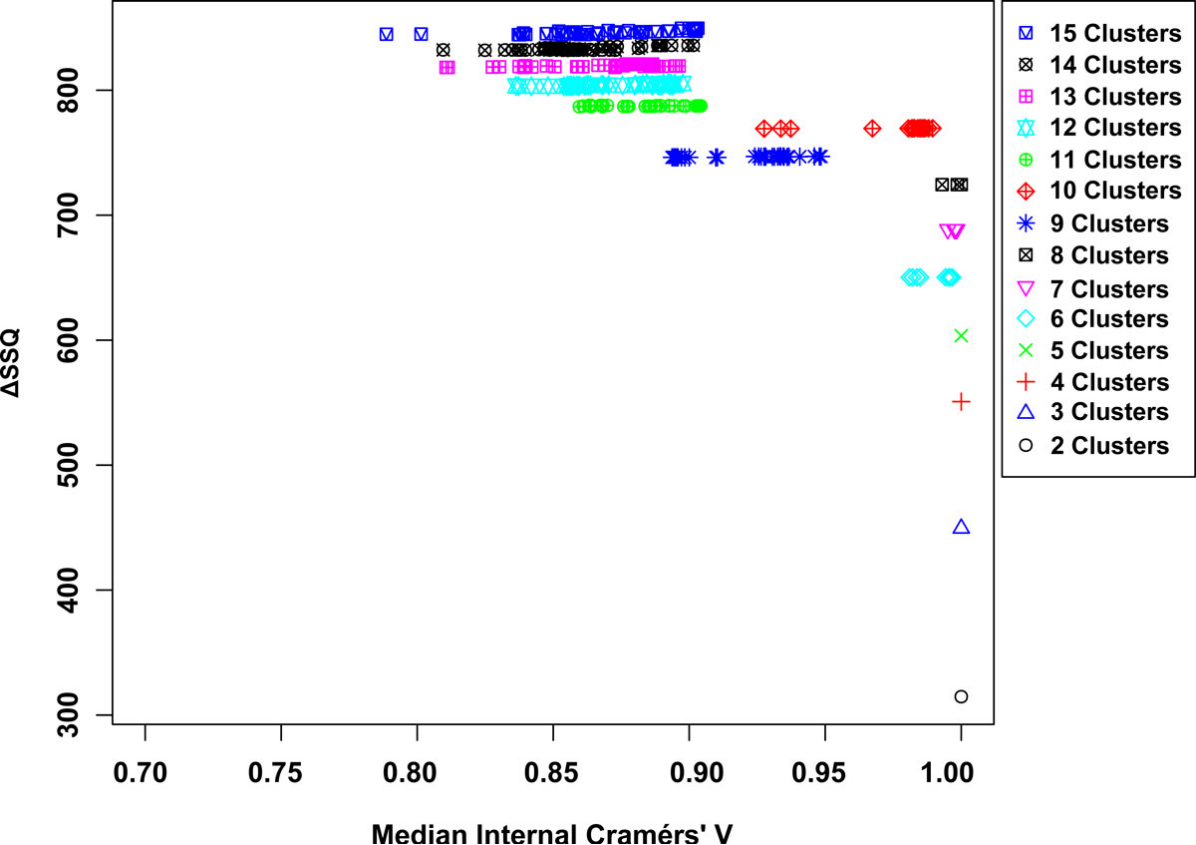
**Separation Concordance map for cardiotocography dataset**. Full Separation-Concordance map for the cardiotocography data highlighting the ΔSSQ on the y-axis and the internal median Cramérs' V on the x-axis. For 500 initialisations of the k-means algorithm for each value of **k**.

Applying the framework gives the SeCo map shown in Figure 3, upon inspection it is clear that for low numbers of clusters, **k **= 2 to **k **= 5 there is minimal variation between the top 10% of solutions. For values of **k **between 6 and 8, there is still minimal variation in the solutions, although the six cohort solution separates into two similarly performing groups. Increasing **k **further, we can see for **k **= 9, there is substantial variation and a drop in the relative performance of the solutions, although this recovers for **k **= 10, the natural number of solutions. Beyond this a continued drop is seen in the overall performance of solutions in conjunction with increasing variance.

### Thresholding the objective function

**Figure 4:**
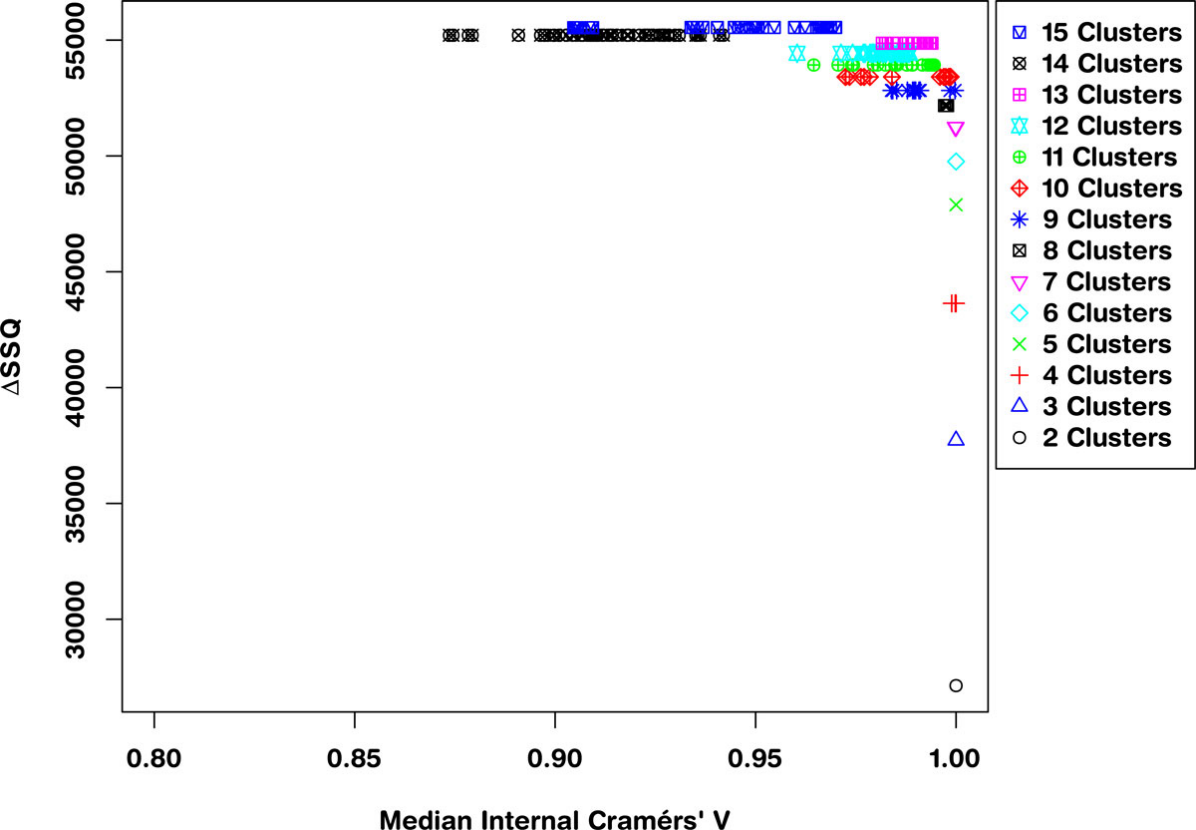
**Separation Concordance map for synthetic dataset using 10% threshold for separation metric**. Separation-Concordance map for the synthetic data, highlighting the top 10% ΔSSQ on the y-axis and the internal median Cramérs' V on the x-axis. For 500 initialisations of the k-means algorithm, highlighting 50 for each value of **k**.

Applying a threshold to the ΔSSQ values, such that only the top 10% of values are considered, i.e. the best separated solutions, gives the SeCo map as shown in Figure 4. This shows that even within the top 10% of values based on the total Within Cluster Sum of Squares, there are large variations in the solutions, as **k **goes above 8 partitions. More importantly, these values of SSQ are largely the same, so the selection of a best solution using SSQ as a metric alone is prone to variation when repeated.

**Figure 5:**
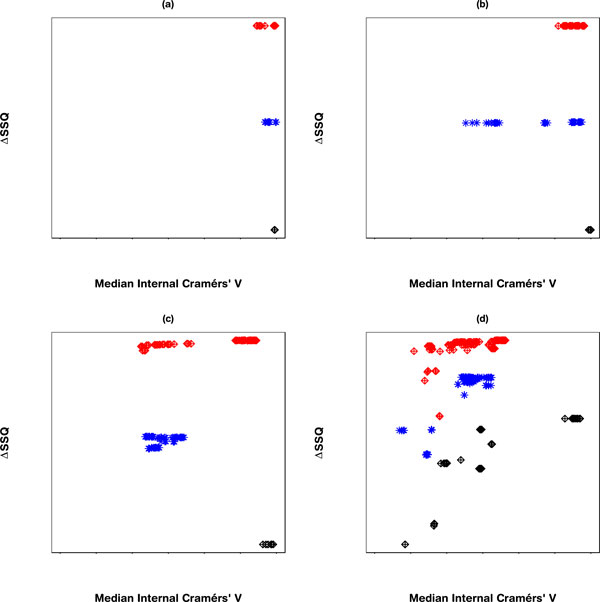
**Separation Concordance maps using varying thresholds for synthetic data**. Separation Concordance map for three values of **k**: 8, 9 and 10, respectively with varying thresholds. Figure: (a) Top 10% of solutions, (b) Top 30% of solutions, (c) Top 70% of solutions, and (d) No threshold. The y-axis represents ΔSSQ and the x-axis the median internal Cramérs' V.

Evaluating the SeCo map with the objective of selecting a potential solution, it appears that the best candidate solutions are k = 8, k = 9 and k = 10, based on their high internal consistency (all having a median CV > 0.95), and the solutions being tightly grouped together in the map. To highlight the importance of thresholding, Figure 4 concentrates on these three partition sizes: Figure 5(a) showing a threshold of 10%, 5(b) 30%, 5(c) 70% and 5(d) no threshold. Inspecting the four maps, it is clear that using a threshold exposes the true underlying partitions of the data, evidenced by the improving consistency in the maps as the threshold is tightened.

**Figure 6:**
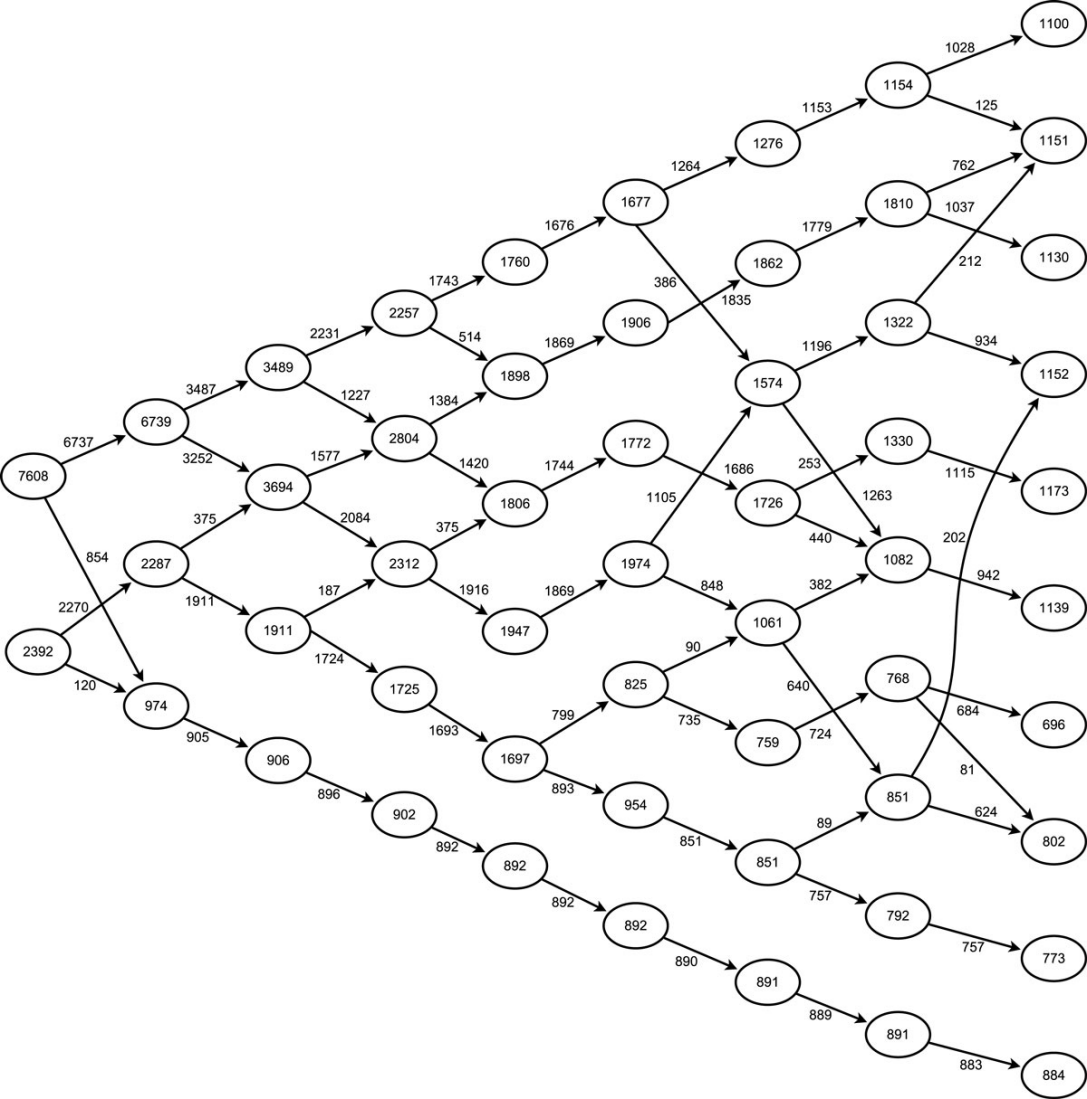
**Partition tree highlighting inter-cluster structure**. Hierarchical chart of cluster solutions, arrows show movement of data points within the cohorts as the value of **k **increases (from left to right), lines were not indicated where the movement comprised less than 10% of the originating cohort, for clarity purposes.

Having identified internally consistent partitions of the data for each value of **k**, it is now possible to trace a pathway through the solution space to visualise the structural stability of the data. Figure 6 shows the partition tree of the solutions for k = 2 to k = 10, where the movement of the data between different partitions is observed, allowing the changing structure of the data as **k **increases to be visualised. The following observations are noted:

• The well separated cohort (number 8 in Figure 1) separates from the other cohorts as early as the three partition solution, and remains separated in all solutions.

• For values of **k **below 7, the cohort structure is stable, whereby an increase in **k **results in one or more existing cohorts dividing to produce the new cohort.

• For partition sets with values of k higher than 8, there is substantial intermixing of the cohorts

Given the strong mixing between the original cohort pairs, these results are consistent with the original cohort structure, as described in Figure 1.

### Benchmarking results

The benchmarking was performed to compare the reproducibility of SSQ alone with that of the SeCo framework, using two measures of performance, as the underlying data becomes increasingly sparse. This is achieved by applying k-means to each of the five artificial datasets (10000, 5000, 2500, 1000 and 500 data points, respectively) and the Cardiotocography dataset 500 times, selecting the solution with the best SSQ and the solution that the framework highlights as being optimal. Solutions for **k **= 8, **k **= 9 and **k **= 10 were selected and compared against the reference partitions previously generated. This process was repeated 100 times, such that the reproducibility and stability of each method could be compared.

Three indicators are used to evaluate the dual measure against the single measure approach, the Cramérs' V statistic is calculated for each of the solutions against a reference partition, which gives a performance measure. The accuracy of the clustering is calculated using the proportion of correctly classified results, and finally a stability indicator, Affinity is calculated, which provides information about the frequency with which individual data points swap cohorts.

**Figure 7:**
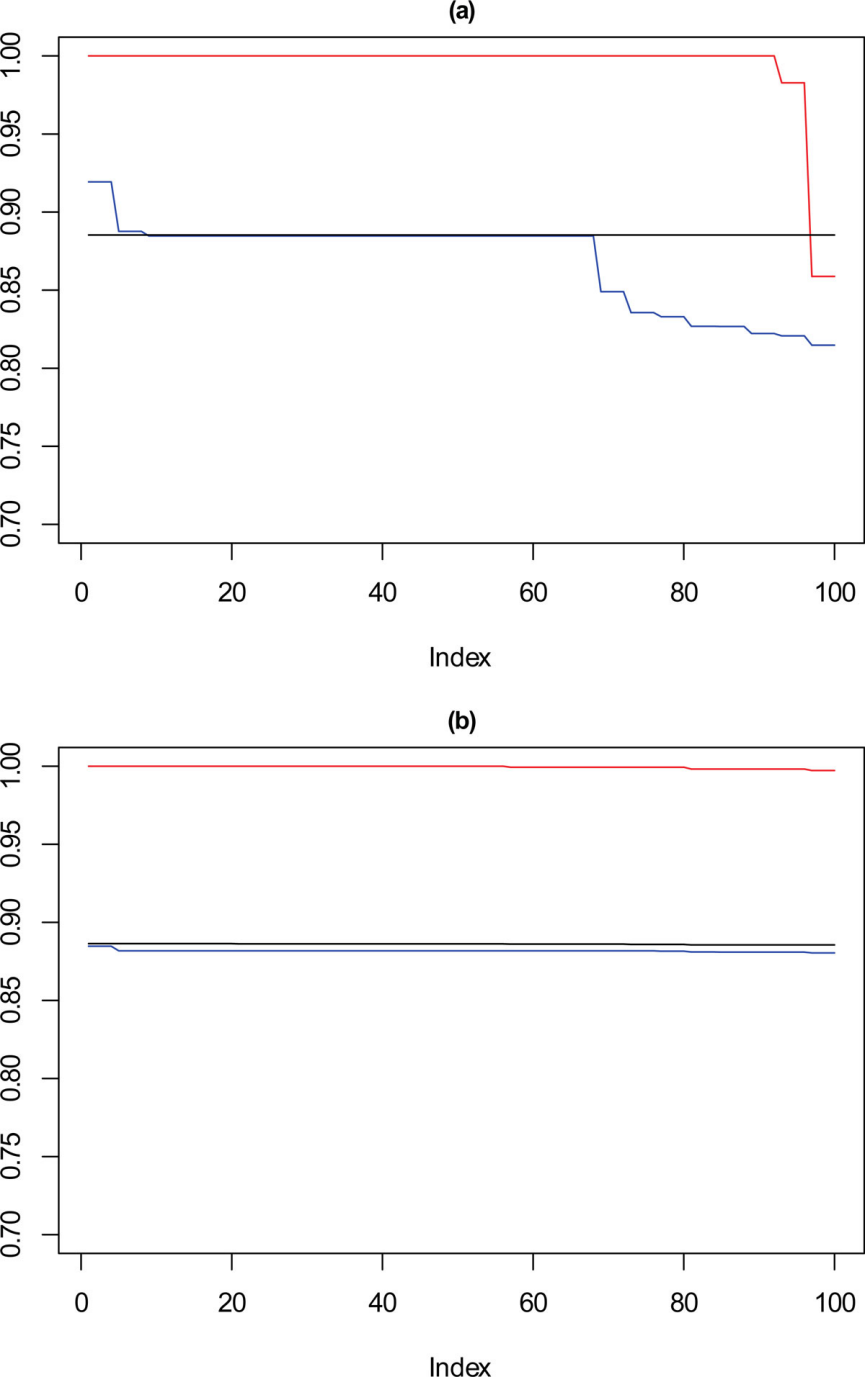
****Performance chart for 10,000 dataset****. Ordered Cramérs' V for 100 runs of the SeCo framework with selection of the best solution being (a) SSQ only and (b) Median Internal Cramérs' V for the top 10% of solutions. Results for the dataset with 10,000 data points. Red lines highlights **k **= 10, blue lines **k **= 9 and the black lines **k **= 8. Confidence intervals for **k **= 8 (Upper: +0.007, Lower: -0.008) **k **= 9 (Upper: +0.006, Lower: -0.007) **k **= 10 (Upper: +0.006, Lower: = -0.007).

Figure 7 shows the performance of SSQ alone (Figure 7a) and the result from the framework approach (Figure 7b), for 10,000 data points, on each plot the red line corresponds with **k **= 10, the blue with **k **= 9 and the black with **k **= 8. These values are the Cramérs' V statistic against the reference partition derived earlier. Confidence intervals are not applied in the plot for clarity purposes, however upper and lower bounds can be seen in the description for each plot.

These show that for k = 10, SSQ performs well, obtaining near perfect concordance with the reference partition, however in approximately ten per cent of cases, it performs less well, and instead of having concordance of ≈ 1, it is possible for the concordance to drop to ≈ 0.85. For k = 9, a more marked variation in the results occurs, with the best case obtaining ≈ 0.925 in 5% of cases, for approximately 30% of solutions the concordance drops to between 0.8 and 0.85, with the remainder settling at ≈ 0.875. For eight partitions there is little variation in the concordance with the reference partition for most results. This is expected as the SeCo map indicates that for **k **= 8 the solutions are highly consistent.

Using the SeCo Framework, in Figure 7b, the performance profile is different, with the solutions exhibiting high levels of consistency throughout. For **k **= 9, all the solutions perform equally well, and whilst there is no longer the higher peak of 5% of solutions, neither is there the dramatically reduced concordance for 30% of results. **k **= 8 shows the same concordance as before, which again is to be expected and corresponds with the amount of variation between solutions indicated by the SeCo map. For **k **= 10, the results are again consistent, however not showing a drop in concordance in the last 5% of cases seen before.

**Figure 8:**
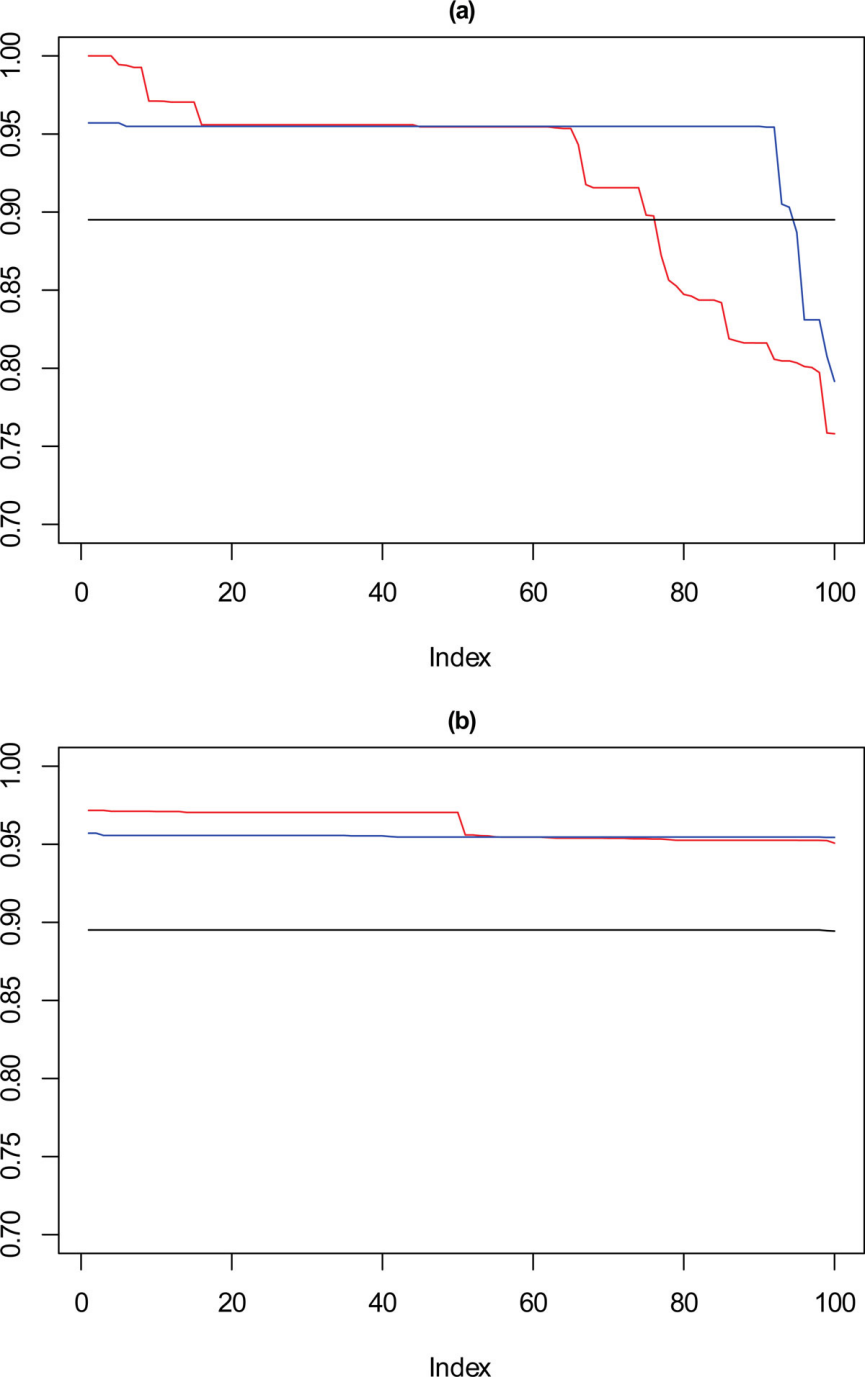
****Performance chart for 5,000 dataset****. Ordered Cramérs' V for 100 runs of the SeCo framework with selection of the best solution being (a) SSQ only and (b) Median Internal Cramérs' V for the top 10% of solutions. Results for the dataset with 5,000 data points. Red lines highlights **k **= 10, blue lines **k **= 9 and the black lines **k **= 8. Confidence intervals for **k **= 8 (Upper: +0.009, Lower: -0.011) **k **= 9 (Upper: +0.009, Lower: -0.011) **k **= 10 (Upper: +0.001, Lower: = -0.008).

Figure 8 to Figure 11 show the same plots for datasets of size 5000 to 500 respectively, showing how the performance against the reference dataset applies as data density is reduced. Similar patterns are shown here where using a single measure for selecting the best solution shows variation against the reference partition not observed with the solution selected using a stability measure as well as a separation measure.

**Figure 9:**
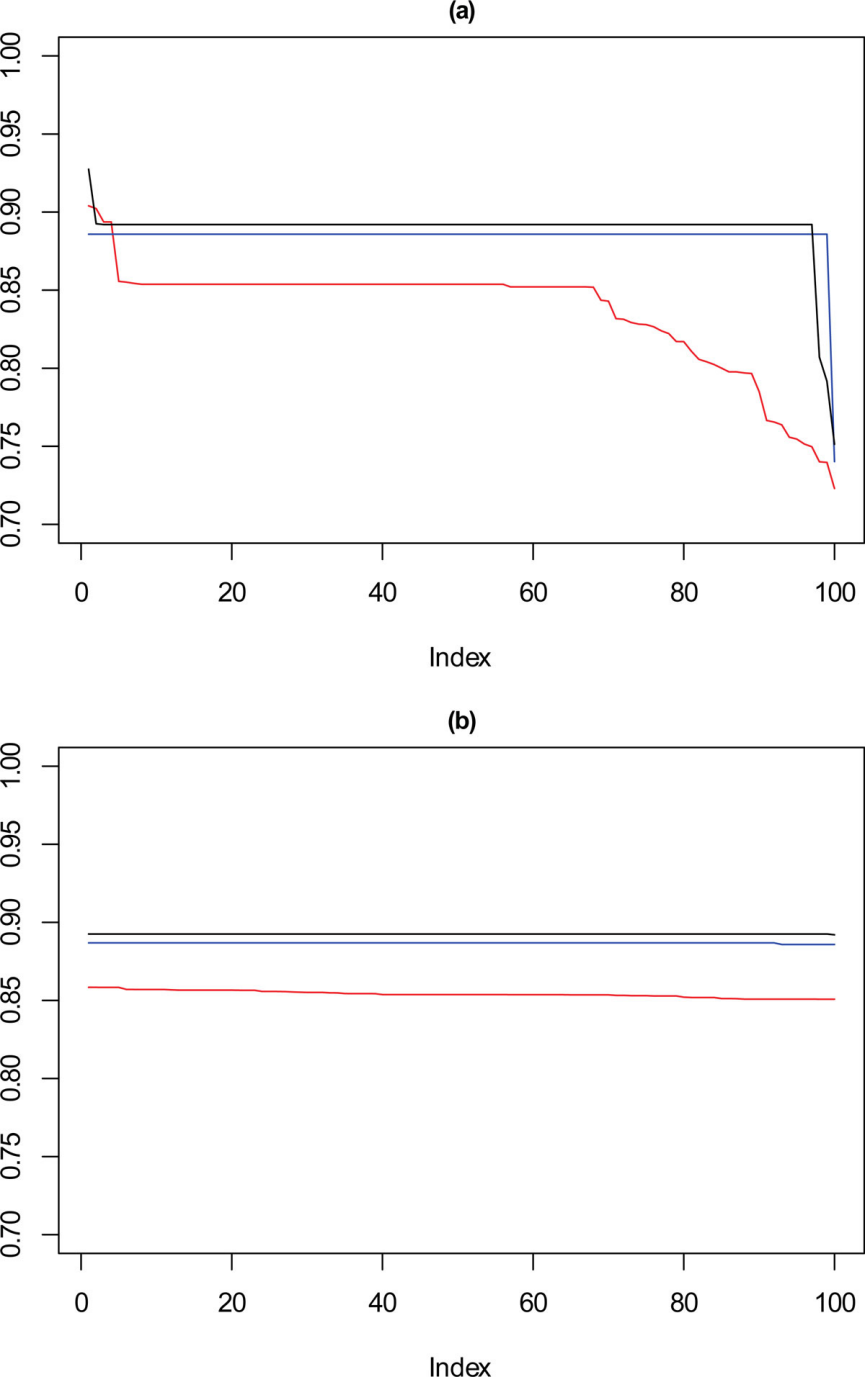
**Performance chart for 2,500 dataset**. Ordered Cramérs' V for 100 runs of the SeCo framework with selection of the best solution being (a) SSQ only and (b) Median Internal Cramérs' V for the top 10% of solutions. Results for the dataset with 2,500 data points. Red lines highlights **k **= 10, blue lines **k **= 9 and the black lines **k **= 8. Confidence intervals for **k **= 8 (Upper: +0.014, Lower: -0.019) **k **= 9 (Upper: +0.013, Lower: -0.018) **k **= 10 (Upper: +0.012, Lower: = -0.017).

For 5000 and 2500 data points as shown in Figure 8 and 9, the observations continue to hold, with there being a degradation in the performance of those solutions selected using just SSQ when compared to those selected with a stability measure.

**Figure 10:**
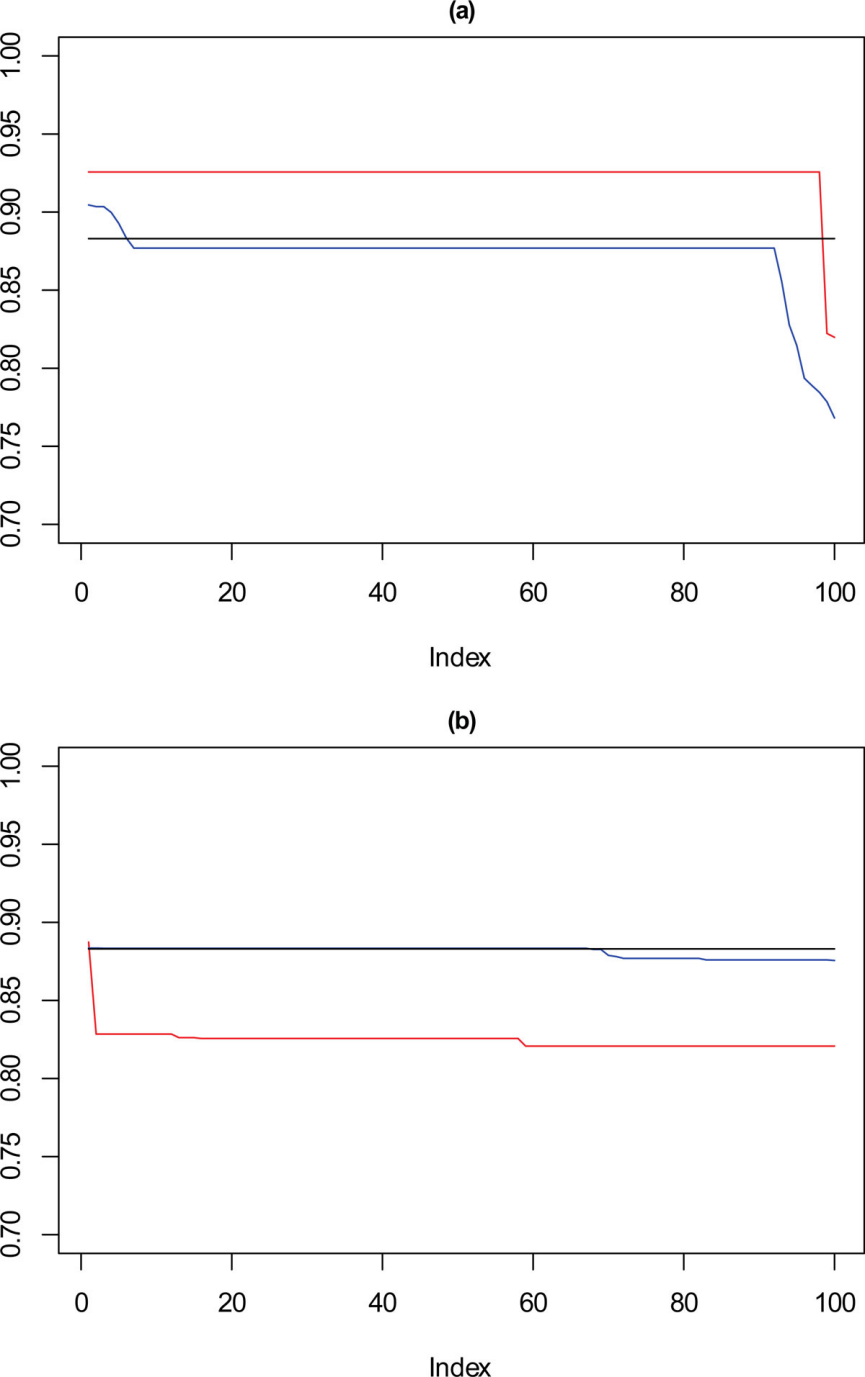
****Performance chart for 1,000 dataset****. Ordered Cramérs' V for 100 runs of the SeCo framework with selection of the best solution being (a) SSQ only and (b) Median Internal Cramérs' V for the top 10% of solutions. Results for the dataset with 1,000 data points. Red lines highlights **k **= 10, blue lines **k **= 9 and the black lines **k **= 8. Confidence intervals for **k **= 8 (Upper: +0.018, Lower: -0.029) **k **= 9 (Upper: +0.017, Lower: -0.027) **k **= 10 (Upper: +0.016, Lower: = -0.026).

**Figure 11:**
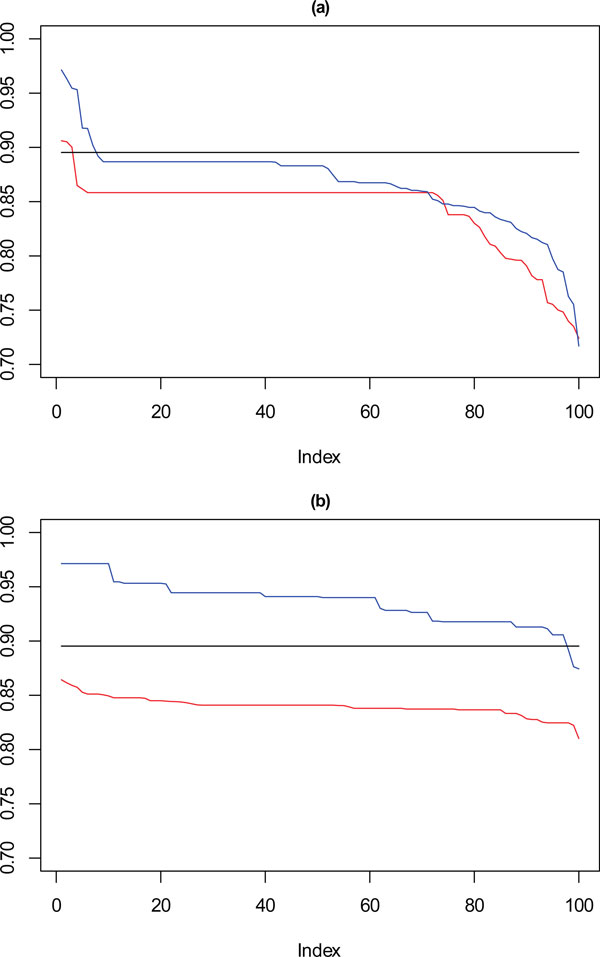
****Performance chart for 500 dataset****. Ordered Cramérs' V for 100 runs of the SeCo framework with selection of the best solution being (a) SSQ only and (b) Median Internal Cramérs' V for the top 10% of solutions. Results for the dataset with 500 data points. Red lines highlights **k **= 10, blue lines **k **= 9 and the black lines **k **= 8. Confidence intervals for **k **= 8 (Upper: +0.023, Lower: -0.043) **k **= 9 (Upper: +0.022, Lower: -0.044) **k **= 10 (Upper: +0.020, Lower: = -0.042).

In Figure 10, for the 1000 point dataset, where **k **= 9 there is a drop in stability for approximately 10% of solutions, not seen for the solutions chosen by the framework. For **k **= 10, solutions selected by SSQ exhibit a stable profile, but with higher concordance than the solutions from the framework. Figure 11 shows that with a very sparse dataset of 500 points there is a clear difference between the performance of the solutions for **k **= 9, which is the reverse of the observation for **k **= 10 with the 1000 point dataset. For SSQ, over 90% of the results are below 0.9 concordance, with the framework solutions, having over 90% of results above 0.9 concordance.

**Figure 12:**
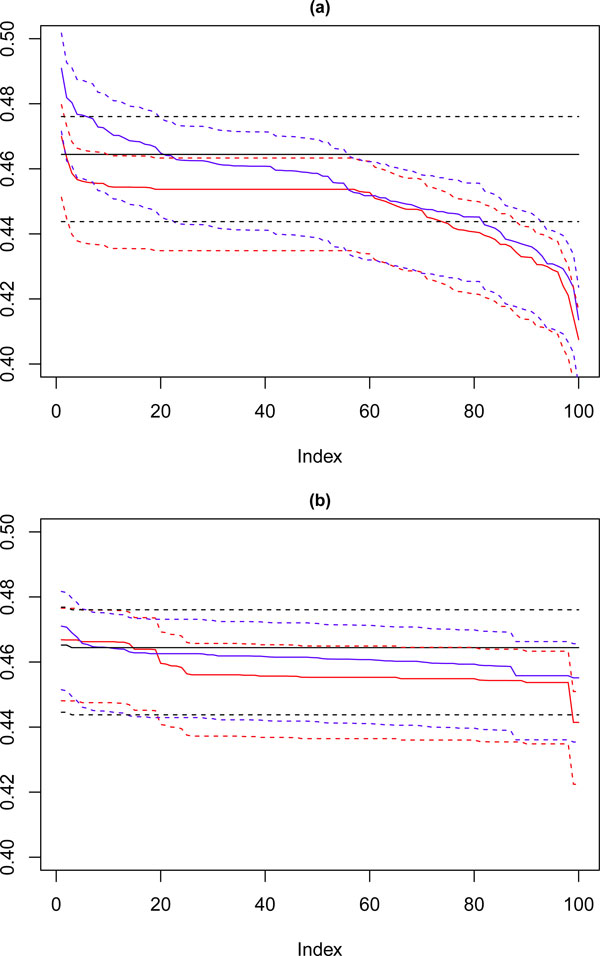
****Performance chart for cardiotocography dataset****. Ordered Cramérs' V for 100 runs of the SeCo framework with selection of the best solution being (a) SSQ only and (b) Median Internal Cramérs' V for the top 10% of solutions. Results for the dataset with 500 data points. Red lines highlights **k **= 10, blue lines **k **= 9 and the black lines **k **= 8. Confidence intervals for **k **= 8 (Upper: +0.009, Lower: -0.011) **k **= 9 (Upper: +0.009, Lower: -0.011) **k **= 10 (Upper: +0.001, Lower: = -0.008).

Looking now at the cardiotocography dataset, the same method of visualisation is used to assess performance of the solutions, where each of the 100 iterations is compared against the known partition of the data. The results for this are shown in Figure 12 where again the solutions for **k **= 8, 9 and 10 are visualised, the red line corresponds with **k **= 10, the blue with **k **= 9 and the black with **k **= 8. Figure 12(a) shows the performance of the 100 chosen solutions for the SSQ measure alone, and Figure 12(b) shows the performance for the dual measure approach. As was seen with the artificial data, the dual measure approach has a more stable profile, and whilst there is deterioration of the solutions beyond the top twenty solutions, this is a step change of approximately 2%, when compared to the drop of roughly 6% for the single measure approach.

Of note is that there is a continuous degradation in performance for this dataset for each value of **k **for the single measure whereas for the dual measure the profile is far more stable. For both sets of solutions **k **= 8 has a flat trajectory and produces largely the same solution, and it is interesting to note that for the SSQ values approximately 20% of both the **k **= 9 and **k **= 10 solutions would fall below the lower confidence interval for this line, unlike the dual measure where only the **k **= 10 line would drop below this level and then for much less than 5% of solutions.

Table 3 shows the accuracy and affinity for each of the six datasets mentioned here, the comparisons here are all for the 10 cohort solutions as to measure accuracy the contingency table against the underlying solution must be square. The mean and standard deviation of the classification accuracy for the same 100 runs above are shown along with the mean affinity. Use of the framework should result in an expected improvement in the stability of solutions, i.e. the standard deviation. This is shown to be the case for all six datasets where the standard deviation for the dual measure approach is an order of magnitude lower in four of the six cases, and less than half in the remaining two. Equivalent accuracy is returned in four of the six datasets with an improvement in one, for the 1000 dataset accuracy is lower, but this falls in line with previous observations reported above. Comparable affinity is observed for three of the six datasets, with substantially better results for the cardiotocography and 2,000 artificial dataset.

**Table 3:** Summary results for six datasets comparing accuracy and affinity for the single measure and dual measure framework

	Single Measure		Dual Measure	
Dataset	Accuracy (Std. Dev)	Affinity	Accuracy (Std. Dev)	Affinity
Artificial 500	0.7758 (0.038)	0.922	0.7701 (0.015)	0.931
Artificial 1,000	0.9263 (0.037)	0.994	0.7773 (0.015)	0.980
Artificial 2,000	0.7332 (0.058)	0.888	0.7345 (0.003)	0.992
Artificial 5,000	0.9079 (0.089)	0.937	0.961 (0.008)	0.989
Artificial 10,000	0.9929 (0.032)	0.993	0.9994 (0.001)	0.999
Cardiotocography	0.3655 (0.017)	0.792	0.3775 (0.002)	0.983

Current best practice of using SSQ to select a single k-means partition set from many, is shown here to perform less consistently than might be expected, and repeated application of this metric has significant potential to produce a sub-optimal result. By contrast using a stability measure in conjunction with SSQ has been shown to perform consistently and aside from a particular result, the pattern is stable, in that using the stability measure in conjunction with the separation measure improves the stability and reproducibility for obtaining a solution when using k-means. In eleven of the twelve benchmark comparisons the SeCo framework performed equivalently to or better than selecting the solution with the lowest SSQ alone.

## Conclusions

A framework is proposed for combining two performance measures, one for intra-cluster separation and the other to measure inter-cluster stability, which guides the sampling of a single partition after repeated random initialisations of the standard k-means algorithm. It is shown that mechanistic application of the proposed method returns very well associated cluster solutions for each cluster number, especially relevant for data where well-separated cluster partitions can show weak association, reflecting poor correspondence in the sense of a contingency table comparing cluster membership, quantified by the concordance index, among clusters with high values of the separation index.

A bioinformatics and multiple synthetic datasets have shown that the sampled solutions are consistently good in their agreement with the known recoverable data structure. Repeated application of the framework to both the synthetic and real-world data show that the performance of the dual measure approach in general better than that of a single metric. This illustrates the main contribution of the paper, namely to show how consistently good clustering solutions can be sampled from the local minima generated by repeated random initialisation, through the use of a visualisation map of the relative performance of each local minimum compared with the rest. The result is to move away from the currently accepted reliance on optimal cluster separation alone, since this can result in unnecessary variation in cluster composition as measured by the cluster stability measures and, furthermore, is shown empirically to be less associated with the known data structure.

Of great importance for bioinformatics is the need for consistent assignment of individuals to a single cluster, the dual measure approach shows a greater likelihood of obtaining stable partitions both in terms of gross structure and also the affinity of individual elements to particular cohorts.

The correlation of co-occurrence of score pairs in the SeCo map with useful choices of cluster number confirm the merits of the stability-based principles outlined in [17] but refines and extends the definition and application of this approach. It is conjectured that the framework will extend to generalised k-means procedures, including the use of medoids for continuous data and k-modes for discrete data.

Further work will use pairwise concordance measures to identify core and peripheral cluster composition, with the aim of obtaining quantitative measures of confidence in cluster membership similar to silhouette diagnostics [25] but without the need for a parametric density-based approach. And also to evaluate selection of optimal **k **within the structure of the framework compared to existing methods such as the gap statistic[36].

## Competing interests

The authors declare that they have no competing interests.

## Authors' contributions

TAE developed the framework concept, coded original software for proof of concept and contributed to the design of the experiments. SJC developed software further, performed experiments with data and contributed to the manuscript. IHJ developed the framework concept, was involved in the design, implementation and analysis of experimental results and contributed to the manuscript. PJGL developed the framework concept, was involved in the design and analysis of experimental results, and contributed to the manuscript. All authors read and approved the final content of the manuscript.

## Declarations

The publication costs for this article were funded by the corresponding author's institution.

This article has been published as part of *BMC Bioinformatics *Volume 14 Supplement 1, 2013: Computational Intelligence in Bioinformatics and Biostatistics: new trends from the CIBB conference series. The full contents of the supplement are available online at http://www.biomedcentral.com/bmcbioinformatics/supplements/14/S1.
